# Therapœia

**Published:** 1825-07-01

**Authors:** 


### THERAPEIA.

Pharmaca nulla valent, nisi quæ sint commoda causis.

1. *Acute Dysentery.** The effects of rapid atmospherical vicissitudes in producing diseases, especially of the class profluvia, and the power of crowding and want of ventilation in rendering these diseases afterwards contagious, have lately been exemplified in a very striking manner in some of our ships of war. We adverted in a former number to the fever in his Majesty's ship Bann, as exemplifying the latter part of the above position; we have lately seen an authentic document from the Fly sloop of war, shewing that acute dysentery—we mean dysentery attended with fever, may, under peculiar circumstances, assume a contagious character, though originally produced by atmospheric vicissitudes.

The Fly had been employed during the severe winter of 1822 on the Scotch station, whence she was ordered to South America, in February 1823. She arrived at Rio, on the 12th April, where the temperature averaged 79°. At Bahia, where she touched, it was at 81. On the 12th June she was off Cape Horn, where the average temperature was 39°, with sleet and snow. On the 11th July, she arrived at Valparaiso—average temperature 59°. Arrived at Callao on the 24th July, temperature 64°. Thus, in a very few months the ship's crew had been subjected to two summers and two winters, with all their intermediate variations. The consequences were such as might be expected—64 cases of dysentery—11 of diarrhœa—and seven of synochus. The dysentery was very severe, and latterly accompanied by typhoid fever, evincing strong proofs of a contagious character. It commenced on the 9th of July. Two cases proved fatal. It spread with great rapidity through the ships' company (120 men,) even after the sick were landed on an island in the harbour of Callao.

"The first symptoms, says the surgeon, noticed by the patient, were uneasiness and irregularity of his bowels,—slight chills,—frequent call to stool, with griping. On making known his ailment which was generally on the morning of the second day after the attack, complained of having passed a restless night, was seized about four A. M. with severe griping across the stomach,—which is getting worse, cannot stand erect for the violence of the pain,—has constant call to stool, voiding nothing but blood and the frothy mucus,—great bearing down pain and tender-

* 1. Private information.—2. Mr. Jones's narrative of a voyage to Calcutta, &c.

ness of the abdomen,—urgent thirst,—head-ache,—countenance flushed,—tongue white and tinged yellow towards the back part,—bitter taste,—hot, dry skin, and a full, rather quick pulse. In the worst cases, besides having some of the above symptoms in an aggravated form, there were, great depression of spirits,—prostration of strength,—pain of loins,—pulse exceeding 180, small and sometimes irregular,—surface clammy and a black crust speedily forming on the tongue.

"The treatment commenced by bleeding to the extent of 18, 20, and 30 ounces (repeated in smaller quantities when the pulse indicated that necessity) until the tenderness of the abdomen and head-ache were relieved, or, the patient could raise himself to the erect position without pain; soon after this, a mixture was taken, containing 6 drachms of the sulphate of magnesia and a grain and a half of antimon. tartar.; this, with a few exceptions, excited vomiting, produced several fœculent evacuations, and determined to the surface. In the afternoon 10 grains of the submur. hydrarg., 4 grains of pulv. antimon. and 1 grain of opium, were taken and repeated regularly every sixth hour till salivation took place, which was generally in 50 hours, with a cessation of fever, and all symptoms mitigated; debility and looseness of the bowels remaining. In the worst cases I gave the submuriate in scruple doses with good effect. After ptyalism was established, the biliary secretion, in many instances, continued irregular and vitiated, causing a return of the tormina and tenesmus; here the mercurial excitement was kept up for some time; for this purpose, the quantity of the submuriate was reduced and combined with the pulv. ipecac. c. opii. The convalescent stage was followed up by the exhibition of the bitter infusion—confectio aromat. with the creta prept.—rhubarb and magnesia in small doses in peppermint water. Latterly I used the tincture of kino and catechu with the tinct. opii, in cases where the secretion of the mucous coat continued acrid and the bowels debilitated, with success.

"In three cases the disease was preceded by costiveness. One in particular (Murray the sub-bay-man,) had the usual symptoms of dysentery, was freely bled and blistered, had taken 120 grains of the submuriate, with occasional doses of the ol. ricini. and sulph. magnes., without producing any evacuations. On the 4th day the abdomen had become tense, he had excruciating paroxysms of pain, and irregular pulse;—he was again bled to 14 ounces; fomentations applied,—enemas were given:—A solution was also taken every half hour of the sulph. magnes. and dilut. sulph. acid,—at the end of six hours the bowels were opened and considerable quantities of black hardened fæces discharged, with hæmorrhage; the pulse became firmer,—severe ptyalism came on, followed by rapid convalescence."

The surgeon observes that, when the system began to feel the influence of the mercury and the disease to yield, the first discharges were generally black, followed by quantities of mucus and blood. These would change to a mottled brownish colour, with fetor and acrimony—then to a bright vegetable green; and ultimately to a natural hue and consistence.

In the two who died, there was inflammation and ulceration of the intestines—especially in the colon and rectum.

The plan above-mentioned is that now almost universally pursued by practitioners between the tropics, and its success forms a striking contrast with that of the old routine of constant purgation. The fever attending this dysentery appears to have been of a typhoid and contagious character—nor need we wonder at this, when we consider the causes which originally produced it, and the circumstances of crowding, &c. by which it was attended.

Before quitting this subject we have to mention, with approbation, a small brochure by Mr. Jones, of Leamington, entitled, "A Concise Narrative, &c. of a Voyage to Calcutta, in the year 1822." The chief diseases which Mr. Jones encountered were purulent ophthalmia, cholera, and dysentery. His treatment of these diseases appears to have been judicious and successful. We can only make room here for one extract relative to the methodus medendi of dysentery, as particularly bearing on the method employed in his Majesty's ship Fly, in an opposite hemisphere of the globe.

"REMARKS.—This was a well marked case of dysentery, and the mere palliative treatment at first adopted, proves how little it availed in arresting the progress of the disease. In short, nothing appeared to have any powerful influence till ptyalism was produced. The scruple dose of calomel was highly useful in removing the tenesmus, and allaying the irritability of the stomach, which I have seen it do in several instances; and willingly I add my humble testimony to the supreme rank which calomel, given in those doses, now holds, of restraining that inordinate action of the stomach, which so distressingly prevails in the majority of tropical diseases, even when all other means have failed. In dysentery, therefore, where it removes the most urgent symptoms of the complaint by its primary operation, and where it subsequently assists in producing that effect on the constitution which is so necessary for the cure; it becomes a remedy of the most vital importance, and cannot be too strongly recommended." pp. 40, 41.

---

2. *Intermittent Affection of the Chest.* Dr. Goupil has published a curious case of this kind, which is deserving of record.

Mons. Th——, 28 years of age, thin, and of delicate constitution, had had frequent attacks of chest affection, which led to the apprehension of phthisis. Having been exposed to cold after severe exercise and much perspiration, he felt himself indisposed for some days, and, on the 28th May, 1824, in the morning, was seized with cough, dyspnœa, and acute pain under the sternum. To these symptoms succeeded an abundant and very fetid perspiration that lasted seven hours, and obliged him to change his linen several times. The perspiration ended, he felt relieved of all the symptoms abovementioned. At seven o'clock in the evening of the same day, he was affected with shivering, succeeded by heat, that lasted till eleven at night—during which period the pectoral affection returned, but not in so severe a degree as before. After this paroxysm he slept tranquilly. Next day he was in perfect health, and congratulated himself on the salutary effects of the perspiration of the preceding day. On the 31st May and on the 2nd June, he had repetitions of the paroxysms, accompanied by incessant cough, dyspnœa, and substernal pain as before. On the 4th June, the paroxysm again returned, and was this day accompanied by a moderate hæmoptysis. The same phenomena occurred on the 6th June, and each of these last two accessions was succeeded by malaise, much debility, and depression of spirits. The cough now continued almost incessantly. The sulphate of quinine was administered in doses of six grains every eight hours, for twenty four hours. The paroxysm of the 8th failed to come on, to the great joy and surprise of the patient. From that period all the symptoms vanished, with the exception of the cough, which continued till the 15th June, when it also ceased, and the patient afterwards continued free from complaint.—*Bibliothèque Medicale, Juillet*, 1824.

---

3. *Oil of Euphorbia Lathyrus, a Substitute for Croton Oil.—Case of poisoning by Euphorbia Peplus.* We noticed, in our number for January last, page 249, Dr. Hufeland's substitute for castor oil, prepared by mixing one drop of croton oil with an ounce of oil of poppy. We have now to notice what is still more important, at least in an economical point of view,—a substitute for croton oil, prepared from an indigenous plant.

Dr. Calderini states, that the great price and difficulty of procuring genuine croton oil, together with the burning sensation it often occasions in the throat, induced him to try the purgative properties of euphorbia lathyrus as a substitute. He procured the oil by expressing the dried seeds, deprived of their husks—fourteen ounces of which gave six of oil. The qualities of the oil thus obtained, after being filtered, are nearly similar to those of castor oil—the colour is the same—it is rather thinner, inodorous, limpid, not acrid, and not disagreeable to the taste. By long keeping and by exposure to heat, it becomes turbid, acquires a dirty colour and acrid taste, and afterwards turns rancid. It burns with a clear flame, without smoke—is insoluble in alcohol—and forms soap with alkalis.

After trying its effects upon his own person, Dr. Calderini administered it to fourteen patients, in the Civil Hospital of Milan; and from these and other experiments he has drawn the following deductions. "The purgative action of spurge oil is certain, strong, and prompt—the dose is small—it is very mild, since it does not occasion vomiting, derangement of the bowels, pain, nor tenesmus; and even in dysentery, arising from intestinal irritation, it operates as pleasantly as pulp of tamarinds."

The purgative power is little inferior to that of croton, and Dr. Calderini prefers it, because its exhibition is not attended by the disagreeable symptoms which the acrid and irritating qualities of the croton oil occasion. The dose to adults is from four to eight drops: to children of two or three years of age, he has given three drops in "pastiles de chocolat." In irritable habits, he has prescribed eight drops in form of emulsion.

It is essentially necessary that the oil be fresh—when rancid, it becomes drastic, and causes griping.

M. Grimaud mentioned, at the meeting of the Royal Academy of Medicine of Paris, held February 21st, that he had repeated the experiments of M. Calderini, and that he considered the spurge oil preferable to the croton.

All writers, from Theophrastus downwards, agree in attributing purgative effects to the different species composing the Linnæan genus Euphorbia: and many plants belonging to the natural family of the euphorbiaceæ, including the croton among others, are known to possess similar qualities.

From Matthiolus, who considers the species under consideration to be the λαθυρις of Dioscorides, cap. CLXI. we learn that, in his time, it was commonly known in the shops by the name of cataputia—a term apparently derived from an Italian appellation, *cacapuzza*, indicating its purgative qualities, (*cacare*, to evacuate—*puzza*, stench) rather than from the Greek, καταπυθω, to have an ill savour. He informs us that the dose ordered by Actuarius was fifteen large, or twenty small seeds: those who wished to be copiously purged, were directed to *chew* the seeds; and those who did not were to swallow them whole. Dioscorides directs only six or seven seeds; his words are "grana (*seeds*, not *grains in weight*) sex aut septem in catapotio, aut cum ficis, aut palmulis, devorata alvum purgant: sed aquâ frigidâ posteâ sorbenda: trahunt autem bilem pituitam et aquas." Old Gerarde quaintly says "the juice of tithymale (spurge) is a strong medicine to open the belly; and, causing vomit, it bringeth up tough flegme and cholericke humours. Like vertue is in the seed and root, which is good for such as fall into the dropsie, being ministred with discretion and good advice of some excellent physition, and prepared, with his correctories, by some honest apothecarie."*

It seems difficult to reconcile the alarming accounts of the violent operation of this and other species of euphorbia, detailed by the old writers, with M. Calderini's statement of the mild action of his oil. Galen and Apuleus speak of emetic qualities, and Matthiolus gives the following passage from Mesue. "Tithymali (euphorbiæ) omnes dejectoriæ facultatis, cordi, jecinori et ventriculo maximè officiunt. Vasa disrumpunt, intestina abradunt, universum excalefaciunt corpus, adeo ut inde facile febres concitentur."† With such authority, well might Ge-

* Gerarde's Herball, by Johnson. London, 1636, folio, pp. 506. Gerarde "was a master in chirurgerie." He lived in Holborn, where he had a large physic-garden, of which he published a catalogue in 1596. He first introduced the euphorbia lathyrus into our gardens.

† Matthioli Commentarii in Dioscoridem, Lugduni, 1562. Folio, pp. 655.

rarde exclaim, "these herbes, by mine advice, would not be received into the body, considering that there be many other good and wholesome potions to be made from other herbes that may be taken without perill."

On the one hand, we see no reason to doubt the accuracy of M. Calderini's statements—indeed, the repetition of his experiments by M. Grimaud is sufficient to prove their correctness. On the other hand, we have known the common petty spurge (euphorbia peplus) prove fatal when taken inwardly, thus confirming the statements of the old writers. It is very probable, however, that the expressed oil of the seeds is much milder than any other part of the plant—and, if so, the apparent discrepancy between the two accounts may be readily explained.

The euphorbia lathyrus, or caper spurge, is a native of the South of Europe, and was first introduced into our gardens, where it is not uncommon, more than two centuries ago. It is naturalized somewhere in Bedfordshire, and in most old herb-gardens. It is figured in Smith and Sowerby's English Botany, tab. 2255—Gerarde's Herball, page 503, fig. 18—and Hill's British Herbal, plate 22. Flowers in May and June. Other species will probably be found to be equally efficacious: of these, the E. Helioscopia (sun-spurge) and E. Peplus, (petty spurge) are common with us in all cultivated places, and certainly deserve trial.

We are enabled to add the case of poisoning, above alluded to, by favour of a correspondent.

A boy, 6 years of age, whilst playing in the fields at the game of "horses" one afternoon, ate, in order to support his assumed character, some herbs, among which the euphorbia peplus was afterwards ascertained to be the only one of noxious qualities. In the evening he became sick, and vomited many times during five or six hours of the night—he was also purged, and had great thirst. The parents, not aware of the cause of his complaint, administered some simple remedies, and did not seek medical advice until the following morning. When I visited him, at half past 8, A.M. there were spasmodic twitchings of all the limbs—pupils dilated—a dark areola around the eyes—small pulse—rather damp skin—hiccup—incapability of swallowing—insensibility. In a few minutes the convulsions ceased—the breathing became hurried, attended with sighing, yawning, and, sometimes, a deep breath—the pupils contracted—the pulse became scarcely perceptible—the hands blue and cold—saliva flowed from the mouth, and some yellowish watery fluid passed involuntarily from the bowels. He died at eleven o'clock, A.M. The body was examined twenty-one hours after death.

*External Appearances.* The joints of the lower extremities rigid—those of the upper ones flexible: fingers bent inwards—their extremities and nails of a dark colour: lips purplish—a slight purplish appearance on the forepart of the abdomen and neck: all the dependent parts of the body of a deep purple colour.

*Digestive Canal and Abdomen.* A small quantity of light green transparent fluid flowed from the mouth on moving the body. Tongue covered with a thick coat of whitish fur, and the papillæ of its root much elevated and highly vascular. Tonsils, fauces, and whole of the pharynx, highly inflamed. *Stomach* distended with air only; the pyloric half smeared with a green substance—apparently digested vegetable matter; the mucous membrane, throughout its whole extent, of a red colour, more or less deep, and appearing raised in numerous small white opaque dots or points. *Small intestines* containing a large quantity of thin yellowish brown fluid, four lumbrici, and a cherry-stone—the mucous coat of a red colour generally—deeply reddened in the lower half, and appearing as if colouring matter had been applied by a small brush. *Large Intestines.* No diseased appearance in the rectum, nor in the cæcum or colon, except here and there a slight vascularity of the mucous coat. No effusion into the peritoneal cavity: mesenteric glands enlarged and minutely injected: peritoneal surface of the small intestines of a red colour, arising from the minute injection of vessels. Omentum more vascular than natural. Stomach injected externally like the small intestines, except that the blood in the vessels did not form continued, but interrupted lines. *Liver* healthy, purple posteriorly, from the *stasis* of blood: gall-bladder of a yellowish green colour externally, and distended with brown bile; all the surrounding parts tinged of a deep yellow colour. Kidneys healthy. Urinary bladder contracted to the size of a man's thumb.

*Organs of Respiration and Circulation.* Rima glottidis much swollen, and, as well as the epiglottis and larynx, highly vascular, and covered with flakes of opaque white coagulable lymph: the larynx and trachea also contained some tenacious green mucus, of which patches were found as low down as the bifurcation of the bronchia. No disease of the lungs or effusion into the thorax. Scarcely a drachm of fluid in the pericardium. A small flake of recent coagulable lymph near the apex of the heart. Right auricle and ventricle filled with polipiform masses and half-congealed blood—left auricle distended with black blood: left ventricle empty. The blood in the veins almost fluid or only partially congealed.

*Head.* Veins of the dura mater and brain distended with blood—about a drachm of fluid in the lateral ventricles. No diseased appearance in the cerebrum or cerebellum. S. C. L.

---

4. *Extraction of Poison from the Stomach.* Dr. Alison relates the following case in the April number of the Edinburgh Journal. On the 5th February, 1825, Dr. A. was called to a gentleman's servant, who confessed he had swallowed an ounce and a half of laudanum, fifteen or twenty minutes before. He appeared as if intoxicated, but not with stupor. Pulse full, strong, and frequent—skin warm—face flushed. Some delay was occasioned in procuring an emetic; but, in a short time Dr. John Campbell arrived with a drachm and a half of sulphate of zinc, which was immediately given in solution. This produced no effect. One drachm more was given, and some tartar emetic, but without benefit. The man was rapidly becoming comatose—his breathing stertorous and irregular. He was roused by dashing cold water on his face, and applying ammonia to the nostrils, &c. Vomiting was not produced till nearly an hour after the laudanum had been swallowed. The ejected fluid smelled strongly of laudanum, but was small in quantity. Profound coma came on. Mr. Bryce's instrument now arrived. Warm water was thrown into the stomach, and drawn out again. It smelled of laudanum. After this process the breathing became more regular. He was stript naked, and cold water was dashed over him. By this he was powerfully excited. He sprang from his seat and fell forwards. He then opened his eyes, and looked wildly about. He fell into an easy sleep, and in the course of the night became again insensible, and seemed at the point of death. He was recovered, though with difficulty. This case shews, first, the facility of washing out the stomach by means of Mr. Bryce's improved instrument. Secondly, the danger of allowing persons under the influence of narcotic poisons to sleep for more than a few minutes at a time, even after the stomach has been evacuated. Third, the great utility of cold affusion in rousing the sensibility, and exciting to action the respiratory muscles.

---

5. *Iodine in Venereal Affections.** Our continental brethren are extending the employment of this new remedy to many other diseases than bronchocele. Scrofulous swellings have been successfully treated in many cases, and it is now employed in syphilitic maladies. M. Richon has published some observations on the efficacy of iodine in blennorrhagy and venereal buboes. In order to guard against the accidents which this remedy occasions in certain irritable constitutions, M. Richon begins with fifteen drops the first day, given in the morning. It is the tincture which he uses. On the second morning he gives 20 or 25 drops—on the third, 30 drops. After this he gives an evening dose, in addition, of fifteen drops, gradually augmenting this last, till 30 drops are taken morning and evening. This quantity is continued three or four days. If no notable effect becomes manifest, and especially if no gastric derangement is complained of, the dose is carried to 40, or even to 50 drops, night and morning. When the dose has arrived to this height, it is common to see the tongue become red and dry, whilst the patient complains of thirst, head-ache, and pains in the bowels, with loose mucous stools. The medium and best dose, according to M. Richon, is 30 drops twice a-day. This is the plan adopted in gonorrhœas, having first premised antiphlogistic regimen—baths—leeches along the urethra, and diluent drinks. The medium duration of the gonorrhœa, under this mode of treatment, is 23 days. In a few cases the author could not go on with the iodine, from some peculiarity, as yet unknown, of constitution.

In the treatment of buboes, M. Richon, after subduing the inflammation, causes friction to be made two or three times a-day, with one or two drachms of the tincture, according to the susceptibility of individuals, and the size of the tumours. After a while the skin of the bubo turns yellow—the epidermis peels off, and the patient feels shooting pains in the part. In general, the swelling begins to diminish in four or five days, and is completely dispersed in eight or ten. In buboes of large size and old standing, a longer period will be necessary for the cure. In these last cases, M. Richon prescribes the tincture internally, at the same time that it is externally applied. In some cases of buboes, as of gonorrhœa, M. Richon has failed with the iodine, and

---

* Eusebius de Salle. Journal Complementaire, Septembre, 1824.

then mercury has succeeded. But we must now come to Dr. De Salle's own observations.

*Case* 1. The Chevalier ——— aged 38 years, had been affected with blennorrhagy for 18 months, during the two first of which, he had suffered sharp pains, difficulty of making water, and sometimes strangury. At the commencement of the third month, he became affected with swelled testicle.* This, by quiet and the usual means, got well enough to permit the patient to go about again. But the discharge from the urethra continued without diminution, even during the testicular inflammation. A swelling of the epididymus remained, and at length became painful, as also the testicle itself. This state had continued many months when Dr. De Salle was consulted. He found the epididymus and right testicle double the size of the others—the testicle itself hard and painful. The patient was terrified at the idea of cancer, and De Salle profited by this terror, in imposing on the patient a more rigid regimen than he had been accustomed to follow. He was confined to the house, and De Salle succeeded in stopping the gonorrhœa by injections of sulphate of zinc, aided by antiphlogistics and emollients. This done, 25 leeches were applied to the scrotum, and afterwards emollient poultices to promote the bleeding and prevent the leech-bites from inflaming. In four days the pains had insensibly diminished, and in five or six, they entirely lost the lancinating character. The use of iodine was now commenced. On the 1st of May, frictions with hydriodate of potash ointment were ordered night and morning on the affected testicle. About the 15th of the month, the tumour began to soften. From the 20th to the 25th, pricking pains were felt in the part, and the frictions were suspended for five days, in favour of baths, lavements, and emollient cataplasms. The iodine frictions were afterwards resumed and continued, and the tincture was given internally. The epididymus has been reduced to the normal size, and the patient is completely cured.

*Case* 2. M. C. a man of letters, 29 years of age, contracted a blennorrhœa in the beginning of April. Inflammation of the left testicle took place on the 23d of the same month, in consequence of sudden suppression of the discharge after drastic purgatives. It was 20 days afterwards, when our author was called in, and found the testicle and epididymus hard and painful—the discharge not having re-appeared. Baths, lavements, 20 leeches — perfect rest — abstinence — diluents. Our author now commenced the tincture of iodine, in doses of twelve drops every morning, which was afterwards increased to double that quantity, and still on to 45 drops a-day. This dose was

* We wonder that Dr. De Salle, whom we know to be an able and classical young physician, should make use of such denominations or phrases as the following, in a *medical* journal. " L'Accident connu sous le nom *populaire* de *chaudepisse* tombée dans les bourses." This is an elegant phrase for inflamed testicle!

not exceeded. By the 11th June, the testicle had regained its natural dimensions; but there was still a little kernel in the epididymus, which also disappeared in three weeks. The patient is completely restored to health.

*Case* 3. M. V. a traveller to a mercantile house, 34 years of age, had had several attacks of syphilis and gonorrhœa, of which he was but badly cured by following the directions of popular pamphlets, or taking the recipes of his fellow travellers. On examination, in the month of May, 1824, M. De Salle found a hardness and enlargement of both testicles, together with a chronic gonorrhœa. After some general means, the frictions of *iodine* were commenced. In six days the integuments of the parts rubbed had become red, with pricking pains. A poultice was now ordered after each friction. By this precaution they were enabled to continue the frictions, and with increased quantities of the ointment. In less than a fortnight the gonorrhœa was reduced to a slight gleet—and in the course of a month the swellings of the testicles were also reduced to one half their former size—at least that of the right, while the left testicle, originally the largest, was greatly softened. By the 28th June, the swellings were nearly reduced, and the *patient* becoming *impatient* of longer restraint, went off to his usual avocations. In this case, the patient had a cluster of warty vegetations at the base of the glans penis, which entirely disappeared during the use of the iodine. Although M. De Salle does not venture to draw any positive conclusion from this circumstance, he evidently anticipates its leading to more important events. He is now determined to give iodine a fair trial in syphilis, as a substitute for mercury. Time will tell the success of this trial; but we venture to predict that, like sulphur in itch, "there is nothing like mercury," in syphilis. In conclusion, we may remark that a perusal of the above cases has not clearly proved to us that the iodine frictions were much more efficacious (considering the other antiphlogistic and discutient measures combined with them) than mercurial frictions would have been, under similar circumstances. We all know how generally these engorgements of the testicles, remaining after hernia humoralis, will be dispersed by leeches, poultices, and weak mercurial liniments, combined with rest, abstinence, and gentle evacuations. Who will argue that such means would not have availed in the cases narrated by M. De Salle, and with equal celerity? By these observations we are far from undervaluing the iodine frictions. Indeed we almost daily see proofs of their utility, in the reduction of glandular swellings. But we think iodine is in danger, like many other preceding good remedies, of indiscriminate application, and undeserved loss of character.

---

6. *Meningitis.** The subject of this case was a weakly child, six-

* Mr. Davies. London. Med. Repos. April, 1825.

teen months old, with fair skin, blue eyes, and large head. He had had several attacks of inflammatory action about the head, which had been subdued by leeches and calomel. There was lateral curvature of the spine. On the 26th November, he was attacked with severe pneumonia, which was subdued in a few days by leeches, blisters, and purgatives. On the 30th, symptoms of enteritis appeared, requiring the usual remedies for that disease. On the 2d December, the head became, in turn, affected. The pupils were contracted—intolerantia lucis—rolling of the head—insomnium—screaming—pulse from 150 to 160, and very weak—strength very much reduced by the preceding attacks and their remedies. The bowels were open, but the stools consisted only of green and black slime. Surface pale, and extremities cold—head hot, with knitting of the eyebrows. The head was shaved, and evaporating lotions applied—warm semicupium—three grains of calomel every three hours. 3d. The same state and remedies. 4th. Worse in most respects. Five grains of calomel and a ninth of a grain of opium every three hours. 5th. Considerably easier. Slept some in the night, and the symptoms are more favourable. Perstat.—By the 9th, the child was much improved, and on that day the calomel was reduced to three grains for a dose, but as the head appeared a little enlarged, half a grain of squills was added to each dose. Under this plan he continued to go on well until the 12th, when a relapse took place. All the symptoms returned as bad as ever. Two leeches were applied to the temples, and five grains of calomel, with a grain of opium, were ordered every three hours. The lotion to be continued—and the blister (which we omitted to mention) between the shoulders, was directed to be kept open. The head continued to enlarge till the 20th December, when it began to be reduced again in size. During this interval (from the 13th to the 24th) the plan of treatment abovementioned was continued. The bowels were open about once in the 24 hours; but as the cerebral disease gave way, the calomel began to act a good deal on the bowels, and to produce griping. The stools were, all the time, green, owing probably, Mr. D. thinks, to the mercury. On the 25th some carbonate of soda was added to the powders. By the 30th, there was scarcely any thing but debility remaining. The meningeal symptoms had entirely disappeared; but the bowels, were a good deal disordered still, and the child was reduced to a skeleton. The powders to be given every six hours, instead of every three hours. A cordial anodyne mixture was ordered. These medicines were continued, with more or less regularity, till the 8th January, 1825. The child was now languid, but the head appeared free from disease. By the 16th, the child was so well as to require scarcely any medicine. By the middle of February, he had gained flesh considerably—" in fact he was quite well." His appetite was now ravenous—his spine much straighter than it was at first. " Between the 26th November, 1824, and 19th January, 1825, a space of seven weeks, this child took sixty-four scruples of calomel; yet it produced no inflammation nor any apparent affection of the gums, nor did any unpleasant symptoms whatever, that could be attributed to mercury, become manifest under its use."

Soon after the above date, the child was suddenly seized with violent bronchial inflammation which terminated his existence in a few days, in spite of every remedy.

*Dissection.* The liver was firmer than usual, and very full of blood. All the other abdominal organs healthy. In the thorax the lungs and pleura were found in a sound state, but purulent matter was observed in the air passages, the lining membrane of which was much inflamed.

" *Head*—As soon as the scalp was removed, we noticed an appearance which we had twice seen before—once very particularly. This was an inflammation of the external table of the cranium. In the present case, the inflammation was on each side of the frontal bone, immediately before the coronal suture. The bone was quite red, and so soft as to be easily shaved with the knife. The inflammation, or, at least, the redness, did not extend beyond the diploë. The pericranium was rather loose over the inflamed parts, but this membrane did not partake of the affection. Whether this affection was produced by the mercury, I am not ready to answer. I have seen it in one case where scarcely any mercury had been taken; but in several other cases, where calomel had been pretty freely given, no appearance of the kind was present. The child was apparently quite free from pain in the head before the attack of bronchitis came on, and had been for several weeks. The dura mater adhered very firmly to the bone all round. This membrane had no appearance of disease. There was no difference in its appearance in the parts corresponding with the inflamed portions of the skull from other parts. The arachnoid was considerably thickened, and was separated from the surface of the brain by a small quantity of serum. The vessels of the pia mater were rather full, but not particularly so. The ventricles contained scarcely any fluid. There was not above an ounce and a half of fluid in the brain altogether, and the principal part of this was between the arachnoid and pia mater, extending down along the spinal marrow." 294.

That the disease, in the first instance, was meningeal inflammation, there can be no doubt, as some of its sequelæ were seen after death by another disease. In such a state of things mercury does not appear to be absorbed, at least in any quantity, which accounts for the inertness of large doses. Indeed we much question whether much smaller doses would not sooner produce the constitutional effects of the medicine.

---

7. *Partial Paralysis of the Face.** Cases of this kind are now divested of many of their terrors, in consequence of the physiological discoveries of Mr. Charles Bell, with which our readers are all well acquainted. These discoveries have clearly proved that the muscles

* Dr. Ed. Delafield. New York Med. and Phys. Journal, Dec. 1824.

supplied by the "superadded nerves" may be paralysed by a disease of those nerves, independently of any affection of the brain itself, or of those parts supplied by the class of "symmetrical nerves"—and that treatment directed to the removal of the disease of the affected nerves will remove the paralysis. Mr. Shaw's cases were, most of them, of such long standing, that he had not an opportunity of testing such treatment. The following cases are interesting on this account.

*Case* 1. N. Van Dine, an American captain, was attacked with rheumatism, in consequence of exposure to cold in the night air, which continued 20 days, in the neck, shoulder, and left hip. At this time he was seized with violent pain in the left ear, and behind the angle of the jaw. The succeeding day he found the left side of the face paralyzed, He was six weeks after this on his voyage home, and was in the same condition when Dr. D. saw him in December, 1822. When tranquil, there was little perceptible, except a slight drawing to one side of the right angle of the mouth. But the moment that he spoke, "the whole face was horribly contorted, and when he smiled or laughed, this took place in a still more extraordinary manner." In either of these actions the left side of the face remained entirely passive, while the muscles of the right side were in full action. The muscles also of the eyelids and forehead were paralyzed—consequently the patient could not close the left eye. It had therefore become inflamed, and the sight impaired. He complained of some difficulty in moving the tongue; and when it was thrust out, it inclined to the right side.—The sensibility of the face remained unaffected. Our author concluded, and with reason, that there was a transference of the rheumatic inflammation to the portio dura of the auditory nerve of the left side. The patient was ordered to be cupped over the origin of the nerve, and then a blister applied. These were repeated and alternated. The captain entirely reovered.

*Case* 2. Miss Adeline B———, aged 17 years, applied to Dr. D. on the 5th March, 1823. A fortnight previously she began to feel a severe pain behind the angle of the jaw, and below the ear on the left side, which gradually increased for a week. At this time she was exposed to a snow storm, and in a few hours afterwards the left side of the face was completely paralyzed. In short, she exhibited all the symptoms detailed in the preceding case. She was cupped, blistered, and had mercurial purgatives. This treatment was persevered in, and she entirely recovered.

Passing over the third case, which is nearly similar to the second, we shall give the fourth in the words of the author.

*Case* 4. "On the 10th May last, I was requested to see Mrs. Mary Ann Ray. Three weeks before I saw her, she was attacked with violent pain in the head and neck, accompanied with severe inflammatory fever. She was bled, and took a purgative, which relieved the febrile symptoms, but the pain in the head continued, varying in violence, with little abatement, for a fortnight. At this time it became fixed in the right ear, and behind the angle of the jaw; it subsided, however, in a few days; and suddenly all the symptoms of partial paralysis of the right side of the face appeared; and in this case to a greater degree than I had yet seen them. The mouth and tongue were so much affected, that she could articulate very indistinctly, and certain words she could not pronounce at all. The eye was widely open, slightly inflamed, and watering profusely, and by the strongest effort it was not possible to close it. In this situation she had been about a week, gradually growing worse. I ordered six leeches to be placed over the trunk of the portio dura, and a blister to be afterwards applied. She was also directed to take gr. ij. of calomel every other night, and ℥ss. of sulph. magnes. on the following morning. In a few hours after the application of the leeches, she was much better; the eye could be half closed, and the mouth was less drawn aside in speaking or smiling. On the 25th, the leeching and blistering were repeated. The improvement was now rapid; and on the 2d June, fifteen days after I first saw the patient, no vestige of the disease could be discovered." 437.

Another case is detailed, as communicated to our author, but does not differ from those that precede.

We some time ago alluded to the case of a physician who was affected in this manner, in consequence of cold caught in the mail coach, while travelling up from Edinburgh to London, on his way to Italy. This case, which was precisely similar to those already stated, was much more obstinate, and did not give way for many months. The treatment was similar to that detailed by Dr. D. This gentleman was under the care of Dr. Johnson and Mr. Shaw in London, and of Dupuytren and others on the continent. He was obliged to have a seton in his neck for some time, before the paralysis gave way.

It is of some importance to know these things, both as regards prognosis and treatment.

---

8. *Puncture—Tetanus—Stimulants.** The patient was an artisan, 51 years of age, who received a puncture from a small nail, in the anterior part of the last phalanx of the thumb, on the 13th of October. On the following day the wound became painful and somewhat tumid. On the third day, there were acute pain and stiffness in the arm, with nausea and vertigo. *Leeches—poultice—purgatives—low living.* This plan was pursued for four days, during which, inflammation along the course of the lymphatics in the arm, with swelling and acute pain up to the axilla, rapidly increased. On the 20th Dr. N. was consulted, there being now rigidity of the jaw, and general stiffness of the affected limb. There was also considerable tremor—slight pres-

* Dr. Nicholls. Med. and Phys. Journ. March, 1825.

sure on any part of the arm excited acute pain, and a peculiar thrilling torture along the radial and ulnar nerves — pulse tense and wiry—100 in the minute—universal lassitude, thirst, and prostration of strength. Apprehending tetanus, the plan of depletion was changed for that of stimulation. Animal food and $\frac{3}{4}$ths of a bottle of sherry wine were allowed that day. He slept for an hour, after dinner, and awoke refreshed. At night, some brandy—40 drops of laudanum — rested much better — slight rigidity of the jaw next morning. Bark and steel tonics, with ammonia, nourishing food, and cheerful society dispersed the tetanic symptoms. Suppuration took place in the thumb—the matter was discharged by free incisions.

Dr. Nicholls bears testimony to the propriety of Mr. Hutchinson's treatment of tic douloureux—a treatment which he says he has pursued for twenty years past, with success. He by no means wishes to deprive Mr. Hutchinson of all honour due for being the first to announce the methodus medendi in question.

---

9. *Purpura Hæmorrhagica.** This case is brought forward by Dr. Belcher, as an illustration of the well-known views of the late Dr. Parry, in respect to purpura hæmorrhagica. The patient was a child $3\frac{1}{2}$ years of age, who was suddenly taken ill on the 21st November, with lassitude, pains in the loins and joints, nausea. To these symptoms succeeded large and repeated discharges of dark fluid blood from the nose, mouth, and anus—the stools exhibiting no fæcal matter. Next came out dark purple blotches on various parts of the body and limbs; these phenomena continuing unabated till the morning of the 23d, when Dr. B. first saw the patient. He found the tongue with a brown fur, interspersed with purple spots, as was the mucous membrane of the mouth and fauces—the pulse 110, feeble and compressible,—the lips blanched—the surface cold and constricted. In compliance with the desires of the friends, and from observing so many symptoms of debility, wine negus and gentle stimuli were administered. But Dr. B. soon had reason to alter his plan, as all the symptoms became exasperated under it. The pulse had risen to 140, full and hard—the surface became hot and dry—the face flushed, with grinding of the teeth, strabismus, convulsive twitchings of the facial muscles, &c. He now ordered the warm bath, which changed the symptoms considerably. A bolus of calomel and rhubarb was given—a turpentine enema thrown up, and repeated till copious intestinal discharges were produced. 24th. Much better; but the bloody discharges and purple blotches continue. The same medicines to be repeated. The oral and nasal discharges have ceased, and the purple spots are fading. Medicines continued. 26th. Every symptom improving. The bath intermitted—the bolus and injections to be continued. 27th. Stools natural, with very few traces of blood. In two days more the patient was convalescent.

* Dr. Wm. Belcher. Med. and Phys. Journal, March, 1825.

